# ID 260 - Construindo um Futuro Sustentável: demandas de saúde e horizontes tecnológicos em imunobiológicos

**DOI:** 10.5327/2237-9622.2025.v34s1.260

**Published:** 2025-11-25

**Authors:** Thaís Pereira Catão, Julianna do Nascimento e Silva Varela, Jenifer Geruza Moraes de Paula, Carla Lemos Gottgtroy, Adriana Danowski, Amanda de Miranda Marques, Beatriz de Castro Fialho, Eliane Matos dos Santos, Elvira Alonso Lago, Maria de Lourdes de Sousa Maia

**Keywords:** monitoramento de horizonte tecnológico, necessidades e demandas de serviços de saúde, indústria pública

## Abstract

**Introdução:**

Estabelecer prioridades na área da saúde é um processo complexo, especialmente nas decisões para melhorar o acesso e prever futuras demandas em saúde. O Instituto de Tecnologia em Imunobiológicos, Bio-Manguinhos/Fiocruz (BM), está revisando seu roteiro tecnológico a partir da integração de conhecimentos entre assistência e indústria, a fim de garantir a melhor estratégia em tratamento, prevenção e diagnóstico frente à matriz de desafios produtivos e tecnológicos em saúde do Ministério da Saúde.

Na primeira fase desse processo, denominada Necessidades em Saúde, foram realizados debates com líderes de opinião da comunidade médico-científica atuantes no Sistema Único de Saúde (SUS) para priorizar as demandas dos próximos dez anos e minimizar os desafios ao acesso universal.

Monitorar o horizonte tecnológico é essencial para identificar tecnologias inovadoras e antecipar demandas que impactem a prática clínica, a organização dos serviços e os aspectos sociais e éticos. A consulta aos especialistas objetivou mapear as perspectivas na área de biotecnologia para atendimento das necessidades de saúde nos próximos dez anos, promover a colaboração com instituições assistenciais e de pesquisa, e garantir a participação social, integrando suas demandas à indústria pública. Apresentamos os resultados gerais do monitoramento do horizonte que constituiu a primeira camada do .

**Método:**

Foram realizadas oficinas nas áreas de pneumologia, doenças infecciosas, oncologia, hematologia, gastroenterologia, dermatologia, reumatologia, cardiologia e neurologia. Em cada uma, um líder científico, pesquisadores e especialistas discutiram as principais doenças e suas demandas de curto, médio e longo prazo. A pergunta norteadora foi "Quais são as tendências em diagnóstico, prevenção e tratamento para atender às necessidades de saúde atuais e futuras com foco em biotecnologia?". Essas reflexões foram traduzidas em produtos e linhas de pesquisa em saúde para análise posterior. Complementarmente, foram coletados dados epidemiológicos e aprofundadas as análises por tipo de tecnologia, status de desenvolvimento e correlação com políticas públicas existentes.

**Resultados:**

Dos produtos identificados com potencial de disponibilização por BM, 43% eram biofármacos, 35% diagnósticos in vitro, 9% vacinas e 13% outras tecnologias. Com relação ao horizonte temporal, 56% eram demandas de longo prazo, 39% de curto e 5% de médio prazo, com 36% de produtos já disponíveis no mercado e 51% de soluções inovadoras. No que tange aos grupos de doenças, a reumatologia apresentou o maior número de demandas biotecnológicas (24%) e a cardiologia teve o menor (4,6%). Entre as demandas biotecnológicas, os anticorpos monoclonais foram protagonistas, representando 24% (160), e, quanto ao tratamento, houve destaque para terapias celular, com 6% (39), e gênica, com 2% (14); CAR-T, com 2% (13); e drogas conjugadas com 1% (10). Enfatizou-se ainda a importância de reconhecer padrões genéticos e ômicos nas doenças que mais impactam a população para medicina personalizada.

**Conclusão:**

A escuta e a avaliação de demandas em saúde são formas de participação social no processo de tomada de decisões. Os resultados encontrados embasarão a discussão institucional para a revisão de produtos e tecnologias. Essa estratégia, aliada a outras fontes de dados, permite um mapeamento integrado e sinérgico que ajuda a definir a melhor ação para analisar produtos biotecnológicos existentes e soluções inovadoras.

